# Substrate Binding Process and Mechanistic Functioning of Type 1 11β-Hydroxysteroid Dehydrogenase from Enhanced Sampling Methods

**DOI:** 10.1371/journal.pone.0025375

**Published:** 2011-09-23

**Authors:** Angelo D. Favia, Matteo Masetti, Maurizio Recanatini, Andrea Cavalli

**Affiliations:** 1 Drug Discovery and Development Department, Istituto Italiano di Tecnologia, Genoa, Italy; 2 Dipartimento di Scienze Farmaceutiche, Università di Bologna, Bologna, Italy; German Cancer Research Center, Germany

## Abstract

In humans, type 1 11β-hydroxysteroid dehydrogenase (11β-HSD-1) plays a key role in the regulation of the glucocorticoids balance by converting the inactive hormone cortisone into cortisol. Numerous functional aspects of 11β-HSD-1 have been understood thanks to the availability at the Worldwide Protein Data Bank of a number of X-ray structures of the enzyme either alone or in complex with inhibitors, and to several experimental data. However at present, a complete description of the dynamic behaviour of 11β-HSD-1 upon substrate binding is missing. To this aim we firstly docked cortisone into the catalytic site of 11β-HSD-1 (both wild type and Y177A mutant), and then we used steered molecular dynamics and metadynamics to simulate its undocking. This methodology helped shedding light at molecular level on the complex relationship between the enzyme and its natural substrate. In particular, the work highlights a) the reason behind the functional dimerisation of 11β-HSD-1, b) the key role of Y177 in the cortisone binding event, c) the fine tuning of the active site degree of solvation, and d) the role of the S228-P237 loop in ligand recognition.

## Introduction

**Figure 1:**
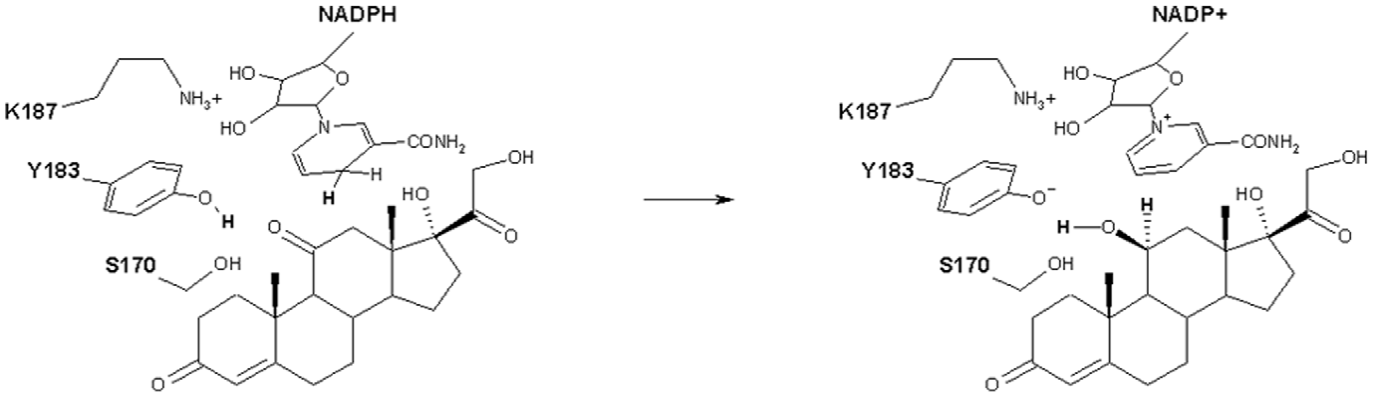
Hypothesised mechanism of action of 11β-HSD-1. The atoms directly involved in the catalysis are highlighted in bold.

Type 1 11β-hydroxysteroid dehydrogenase (11β-HSD-1) is a nicotinamide adenine dinucleotide phosphate (NADPH) dependent enzyme, belonging to the short chain dehydrogenases/reductases (SDR) superfamily [1], [2]. In humans, 11β-HSD-1 plays a key role in the regulation of the glucocorticoids balance by converting the inactive hormone cortisone into cortisol that, in turn, modulates the glucocorticoid receptor [3]. The enzyme is expected to follow a general acid-base mechanism where conserved residues likely important for catalysis comprise S170, Y183, and K187. In the generally accepted reaction model, the tyrosine acts as the catalytic base while the serine helps keeping the substrate in place. The lysine interacts with the NADPH and lowers the pKa of the tyrosine OH, thus promoting the proton transfer. Consequently, the hydride transfer is hypothesised to occur from the C4 of the nicotinamide ring to the C11 position of the substrate cortisone (Figure 1) [4], [5], [6], [7].

The study of the experimentally solved structures of 11β-HSD-1 in complex with inhibitors [7], [8], [9], [10], [11] available at the Worldwide Protein Data Bank (wwPDB) [12] along with experimental data [13], [14] indeed have highlighted important aspects of the protein functioning. However at present, a complete description of the dynamic behaviour of 11β-HSD-1 upon substrate binding is missing. As a matter of fact, X-ray crystallography can ultimately provide snapshots averaged in time and space of the protein motion. On the other hand, mutagenesis experiments deal with alteration of the enzymatic activity (*i.e. K*_m_ values) occurring upon residues mutation. In both cases, the underlying mechanistic causes behind the experimental data are somehow left to interpretation.

To this aim, it is tempting to use computational tools such as docking or molecular dynamics (MD) to gain mechanistic insights at molecular level. Regrettably, some events such as binding/unbinding or folding/unfolding are far from being feasible to simulate within the timeframe allowed by standard processors: in the order of months to simulate hundreds of ns for a protein containing hundreds of residues on ordinary computer systems, not to mention the possible issues originating from the use of some force fields when challenged with long simulations [15], [16], [17]. To overcome these limits, in alternative to the employment of GPU-based clusters [18] or optimised hardware [19], a number of techniques such as umbrella sampling [20], [21], steered molecular dynamics (SMD) [22], [23] and, more recently, metadynamics [24], [25], just to name few, have been devised. The common idea behind these methods is that the evolution of the molecular system is promoted along a number of collective variables that, taken together, account for the event under investigation. Ultimate goal is to overcome free energy barriers that otherwise would limit the energy landscape exploration by trapping the system into local minima [26]. In doing so, one can gather insights from a qualitative and/or quantitative perspective into biologically relevant systems in a reasonable time [27], [28].

In this study, we firstly docked cortisone into 11β-HSD-1 catalytic site and then we used enhanced sampling techniques to simulate its undocking. For the first time, SMD and metadynamics were used in conjunction (and in a sequential manner) to shed light at molecular level on the complex relationship between an enzyme and its natural substrate. Insights into cortisone binding and mechanistic functioning of 11β-HSD-1 are reported.

## Results

### Catalytic pose

A key step when dealing with unbinding simulations is the choice of the ligand starting position within the site of interest. Since an experimentally determined structure of 11β-HSD-1 crystallised with cortisone was not available, we produced such a complex by means of a docking simulation, as explained in the Methods section.

**Figure 2:**
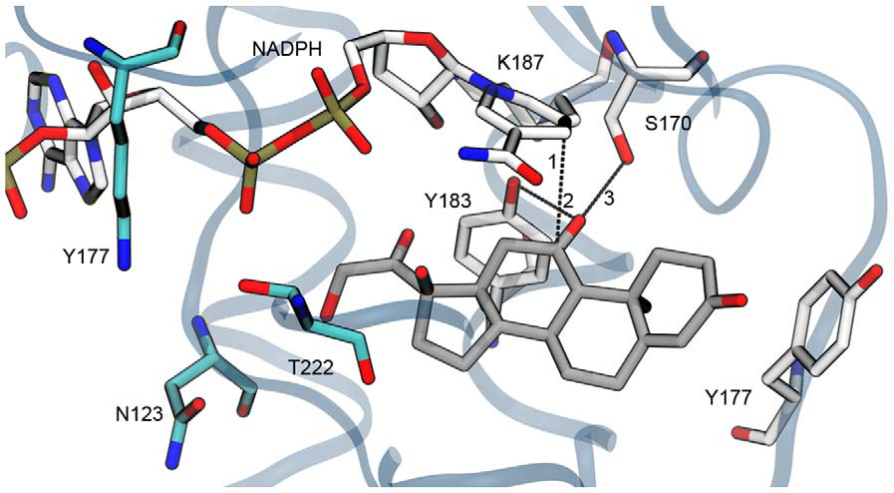
Top ranked pose for cortisone into 11β-HSD-1 binding site. Cortisone, NADPH, and residues likely important for substrate catalysis and stabilisation, are depicted as stick models, C-coloured in gray and in white respectively. K44, N123 and T222 are highlighted as stick models, C-coloured in cyan. The protein backbone is represented as blue transparent ribbon. The dotted lines highlight the distances used to monitor the cortisone stability during the MD simulations (see forthcoming sections).

In Figure 2, the top scoring conformation of cortisone with respect to the selected X-ray structure is reported. On one side Y183 and S170 were within optimal hydrogen bond distance with respect to the carbonyl group of the substrate in position 11. On the other side, the C4 of the NADPH nicotinamide ring stayed right on top of cortisone C11, prone to operate the hydride transfer. Notably, in agreement with the widely accepted catalytic mechanism, in the X-ray model, a conserved lysine (K187) interacted with the NADPH ribose group. This event is expected to induce the Y183-OH positive pKa shift that triggers the proton transfer.

The entrance to the active site could be roughly defined by the residues belonging to two segments, namely S228-P237 and S170-V180, whereas cortisone was placed in a cleft, mainly constituted by hydrophobic/aromatic residues (L126, L171, A172, Y177, V180, Y183, L217, A223, A226, V227, and I230), that was approximately 15 Å deep and 7 Å wide. Conversely, the number of polar residues increased substantially (K44, T220, N123, T124, S125, S170, and T222) at the bottom of the gorge, in proximity of the NADPH cofactor (where the polar part of the substrate resided). In particular, the side-chains of three residues among those above mentioned (K44, N123, and T222) defined a small opening at the bottom of the cleft that might account for the transit of water (see Figure 2). This functional feature that cannot be readily evident from a static snapshot of the enzyme was indeed highlighted during the dynamics simulations of the complex, as described in the forthcoming sections.

Since the 3D arrangement of cortisone found within the active pocket was consistent with the putative catalytic configuration [7], the so obtained complex was subjected to 5 ns of MD simulations to get a reliable starting model for the undocking studies.

### Stability of the enzyme

Taking into account *in vitro* assays, it has been suggested that 11β-HSD-1 minimally functional unit is a dimer [7], [29], [30]. However, the functional reason for the dimerisation has not been fully clarified yet and it remains unclear whether this affects directly the ligand binding process. For this reason, the molecular complex was initially treated as a monomer. We note that in computational simulations of macromolecules, when heavy workloads are required, this is a practice that allows lowering the number of atoms to include in the simulations without affecting, in principle, the overall accuracy [31].

**Figure 3:**
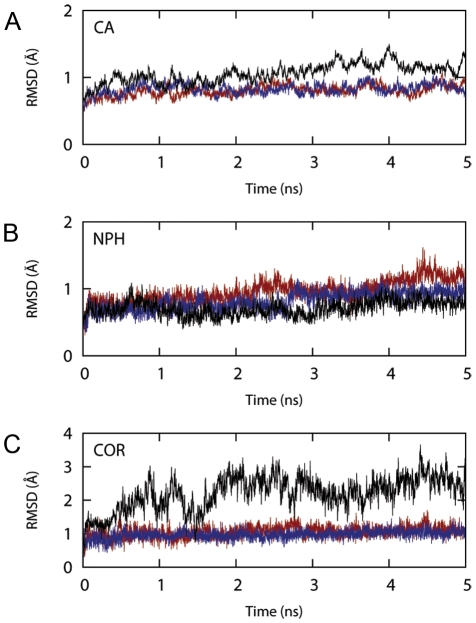
RMSD values during the MD simulation. Proteins Cα (A), heavy atoms of NADPH cofactor (B) and substrate cortisone (C) are plotted as function of time. The black line refers to the simulation conducted on the single monomer. The red and blue lines refer to the simulation of chain A and B of the dimer.

In Figure 3, the Root Mean Square Deviations (RMSDs) of the enzyme's Cα, NADPH cofactor (NPH) and cortisone (COR) during 5 ns of MD are plotted as function of time both for the monomer and the dimer. During the production stage of the MD simulation the monomer's Cα RMSD was consistently higher than the ones of the two chains of the dimer, taken singularly (see Figure 3A and compare the black line, corresponding to the monomer, with the red and blue lines, corresponding to the chain A and B, respectively). Interestingly, the stability of the NADPH cofactor did not seem to be improved by the enzyme dimerisation (Figure 3B) thus producing comparable RMSDs values for all of the three cases. On the other hand, the stability of the substrate cortisone, which resided in the most movable region of the protein, resulted greatly hampered when only the single monomer was simulated (Figure 3C). This recorded behaviour became even more marked during some undocking attempts, where partial protein unfoldings were consistently observed in proximity of the S170-V180 segment.

**Figure 4:**
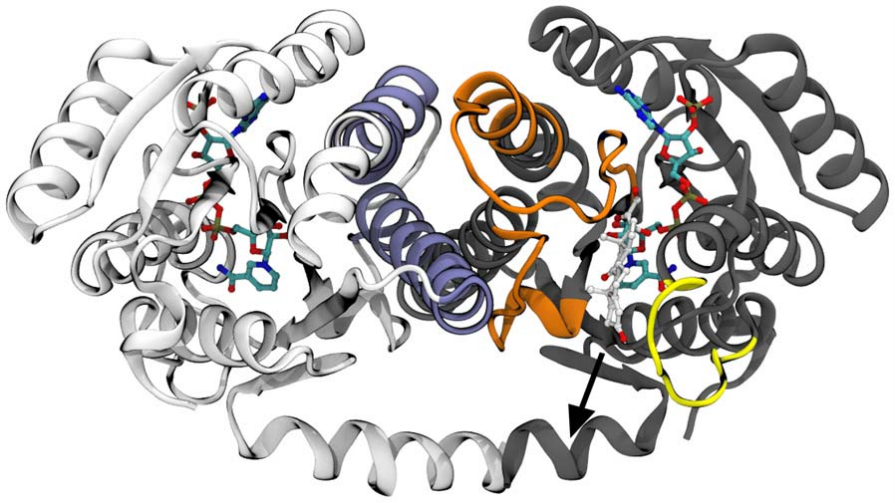
Secondary structure elements directly interacting with cortisone during the unbinding process. H120-N143, S170-V180 residues are coloured in orange while S228-P237 residues are coloured in yellow. For sake of clarity chain A and B are coloured in gray and in white respectively. NADPH and cortisone are depicted as CPK models C-coloured in cyan and in white respectively. The elements of chain B, quenching the displacement of the counter-chain, namely the I133-V149 and S170-V180 segment, are coloured in blue. The black arrow indicates the exit direction of cortisone.

The reason behind such behaviour could be found observing the residues that define the entrance of the active site. The spatial displacement of some of those residues appeared clearly overestimated when the structural elements of the counter-monomer that would naturally constrain their dynamics were missing (see Figure 4).

**Figure 5:**
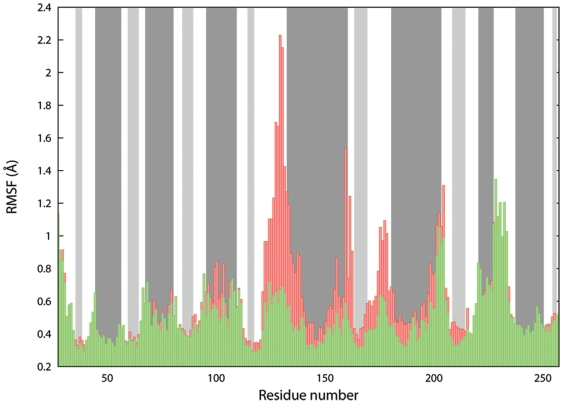
RMSF per residue. The green and red bars refer to the dimer and the monomer, respectively. The background is coloured accordingly to the secondary structure elements found in the starting PDB model (*i.e.* dark gray for α helices, light gray for β sheets and white for unstructured secondary elements).

In particular, the mobility of the regions H120-N143 and S170-V180 was quenched by the residues of the counter-chain in direct contact with them. Also the S228-P237 loop showed a great mobility (see Figure 4 for reference), but this was surprising to a lesser extent, being in agreement with previously reported studies that indicated that loop as highly movable under physiological conditions [7]. In fact, an analysis of the available experimentally determined structures of 11β-HSD-1 highlighted how that loop is likely to assume very diverse conformations, or, in some cases, even to be poorly defined in terms of electron density [7]. Moreover, that particular segment is thought to be evolutionary important for substrate specificity and recognition, being the structurally less conserved region within the SDRs superfamily [32], [33]. In Figure 5, the Root Mean Square Fluctuation (RMSF) per residue, averaged over 5 ns of MD is shown. The plot clearly highlights that even for short time simulations (5 ns), the differences in fluctuations between the monomer (shown in red) and the dimer (the average of the 2 chains is shown in green) were remarkable. Taken together, the above mentioned considerations prompted us to use of the dimeric form to simulate the cortisone undocking (see also the following section).

Even though this finding cannot be *per se* considered conclusive on the subject, overall it offers an explanation on the reason why 11β-HSD-1 minimally operating unit is a dimer. Moreover, it underlines how the unjustified simulation of monomeric forms with the aim to lighten the computational demand can potentially lead to severe artefacts [31].

### Catalytic and substrate stabilising residues

**Figure 6:**
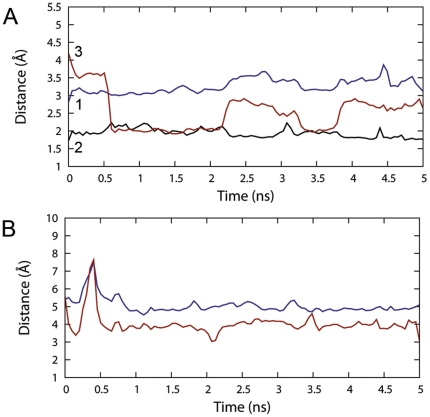
Analysis of the catalytically competent configuration during the MD. (A) The distances used to define a catalytically competent pose are plotted as function of time. For sake of consistency the numbering refers to Figure 2. (B) The distances between the centroids of the cortisone A ring and the Y177 aromatic ring (red line) and the 3-keto group of cortisone and the Y177 oxygen (blue line) are plotted as function of time.

A mandatory requirement for the promotion of the catalytic event is that the position of the substrate within the binding site is compliant with defined geometrical restraints suitably chosen according to the catalytic hypothesis [34]. Although a certain degree of stability of the substrate within the binding site could be already inferred from the analysis of its RMSD (see Figure 3), a convenient way to assess whether the obtained complex was likely to yield catalysis was to monitor the reciprocal spatial arrangement between catalytic residues, cortisone and NADPH during the MD run. In particular we monitored during the MD production run the distances (Figure 6A and Figure 6B) a) between the C4 of the NADPH nicotinamide ring and the C11 of cortisone (blue line in Figure 6A, corresponding to the distance 1 in Figure 2), b) between the 11-keto group of cortisone and the oxygen atoms of Y183, c) S170 (black line and red line in Figure 6A, corresponding to the distances 2 and 3 in Figure 2, respectively), d) between the centroids of the cortisone A ring and the Y177 aromatic ring (red line in Figure 6B), and e) the 3-keto group of cortisone and the Y177 oxygen (blue line in Figure 6B).

The plots illustrate that the distances fluctuations are well behaved and consistent over the entire production time. In particular the catalytically competent pose was stably kept within the whole MD timeframe. Interestingly, the substrate stabilisation due to S170, gained at the beginning of the simulation, was partially disrupted towards the end of the trajectory (although being always within the H-bond range cutoff). The active site regions deviated only minimally from the reference crystal structures, with average RMSD values around 0.5 Å. An interesting aspect of the enzyme functioning came out from the analysis of the dynamics of Y177 with respect to cortisone. Recently, some authors [14] provided a hypothesis, supported by mutagenesis data, that Y177 acts as a substrate stabilising residue through its aromatic ring rather than through an H-bond pattern, as previously thought [35]. In their study, while the Y177F mutant retained the original activity, the Y177A and Y177Q resulted in substantial increases in *K_m_* values for cortisone. In our study, within the MD timeframe, no H-bond was observed between Y177 and cortisone. Moreover, the distance between the A-ring of cortisone and the aromatic ring of Y177 was consistent with a substrate stabilising hydrophobic interaction. To further investigate on the role of Y177, we performed separately the docking simulation of cortisone to the Y177A mutant (see below). It is worth noting that the gradual solvation of the active site, occurring during the early stages of the MD simulations, did not disrupt any of the important interactions between substrate and enzyme already found during the docking simulation.

### Steered molecular dynamics

**Figure 7:**
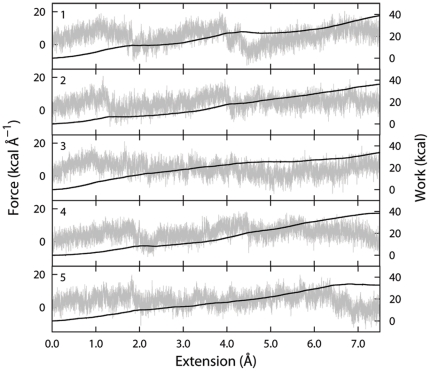
Five uncorrelated runs of SMD to simulate the cortisone undocking. For each run the force (gray line) and the work (black line) needed to bring cortisone from the catalytically competent pose to solution (7.5 Å away) are reported.

To gain a qualitative insight of the unbinding process and to feed the metadynamics protocol with a reliable guess of the exit path, the ligand expulsion was simulated *via* steered molecular dynamics (SMD). Five uncorrelated runs were performed, starting from the last collected snapshot of the MD trajectory, as explained in the Methods section. The runs were monitored in terms of applied force and work needed to pull cortisone out of the active site to solution (see Figure 7).

The differences between the 5 trajectories that could be identified already in the first 2 Å of extension became increasingly pronounced as long as cortisone was being expulsed. This progressively divergent behaviour could be ascribed to the increasing number of possible configurations that the molecule could adopt when moving from a more constrained environment, such as the catalytic pocket, to the solvent, where larger entropic contributions came into play. Being SMD performed at finite velocity, the relaxation of all the relevant degrees of freedom describing the unbinding event is not expected. In fact, in a typical process involving a ligand detaching from a macromolecule such differences are normal and expected. However, some peculiarities common to all the SMD emerged in a couple of key moments, identified both by visual inspection of the trajectories and the analysis of the force and work curves. The first event was the disruption of the stabilising interactions of the catalytically competent pose, occurring within 1 and 2 Å distance from the starting configuration. Overall this event resulted in similar rupture forces for all the studied cases (see Figure 7). However, in runs 4 and 5, the concerted entrance of water molecules from a small opening defined by K44, N123, and T222 (see Figure 2) led to smoother force profiles. The second event was the slide of cortisone over Y177, located at the entrance of the site, on the way to the solvent, occurring within 4 and 6 Å distance from the initial configuration. In this window, the runs 3 and 5 showed a less rugged trend mostly due to the ease of cortisone in leaving the binding cavity and reaching the solvent thanks to favourable hydrophobic interactions with the tyrosine.

The main purpose of the SMD simulations here was to obtain a coarse unbinding path onto which run more refined and time demanding simulations. Nonetheless, taken together the 5 SMD simulations gave a clear qualitative grasp on the process of cortisone unbinding, and partly highlighted some relevant physico-chemical events that could possibly come into play. The run 5, which had the smoothest force profile and the lowest work value, was eventually extended from 1.5 to 3 ns (corresponding to a target extension of 14 Å) and used as input path for the metadynamics study.

### Metadynamics (path collective variables)

A metadynamics simulation was then used to reproduce the cortisone unbinding event and to get a quantitative definition of the free energy surface (FES) along this pathway. In particular the path collective variables (PCVs) protocol was used taking advantage of the low-work trajectory obtained from the SMD simulations. The main advantage of this approach is that, provided a reasonable path, the system is let free to evolve along it exploring all the relevant degrees of freedom. In doing so, the free energy barriers can be conveniently overcome by preserving at the same time the sampling statistics of the chosen statistical ensemble.

**Figure 8:**
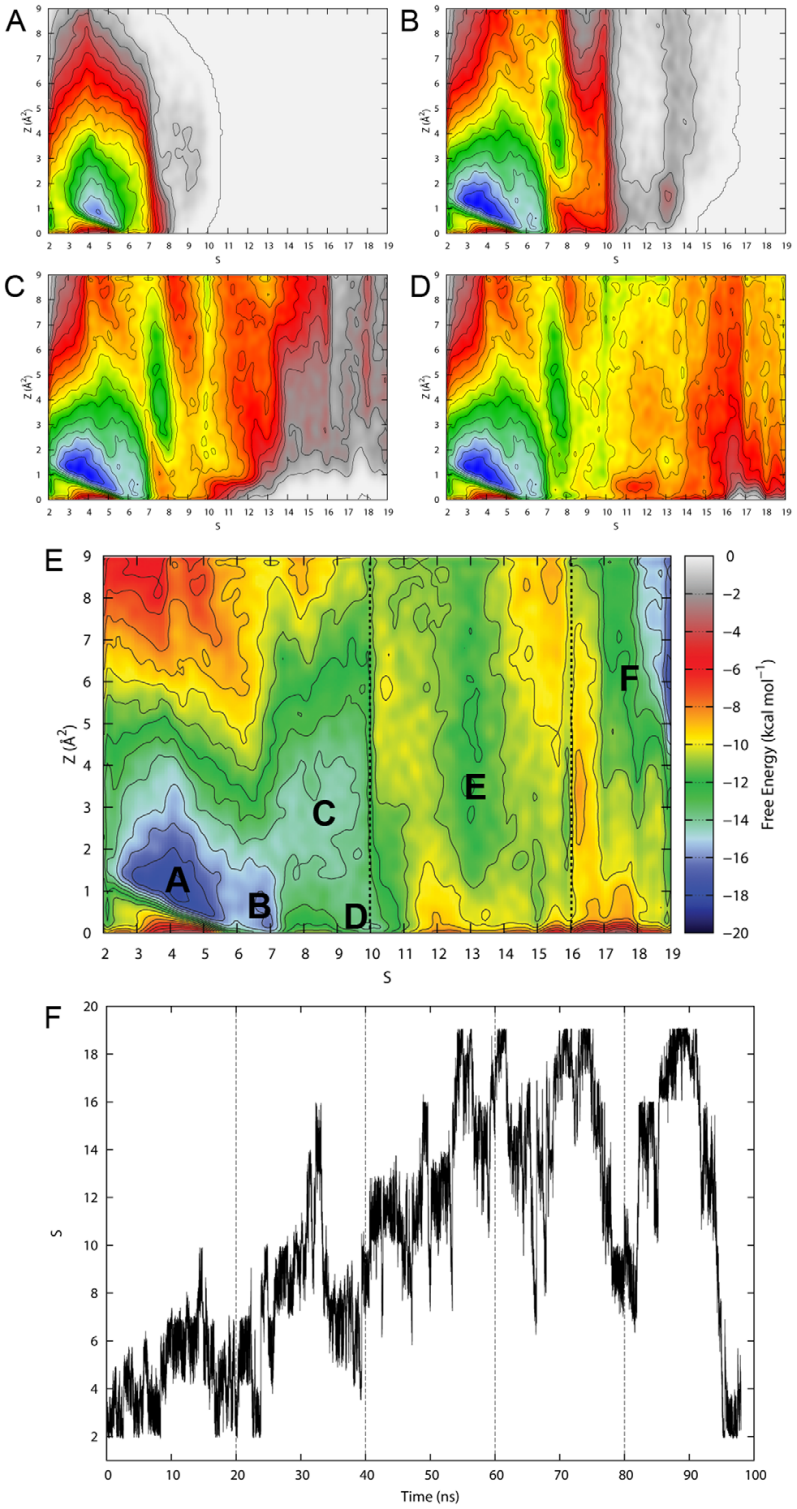
Free energy surface of the cortisone binding process. (A–E) The time evolution of the FES (at 20, 40, 60, 80 and 98 ns) as reconstructed using metadynamics, as a function of *S* and *Z* (see Methods for details). Iso-lines separation is 1 kcal mol^−1^. Worth-analysing minima are highlighted in (E) (labels **A** to **F**). The dashed lines in (E) highlight 3 distinct regions of the surface. The explanation can be found within the text. (F) The variable *S* plotted as function of time. The dashed lines are drawn to highlight the correspondence with the evolution of the FES, (A)–(E).

**Figure 9:**
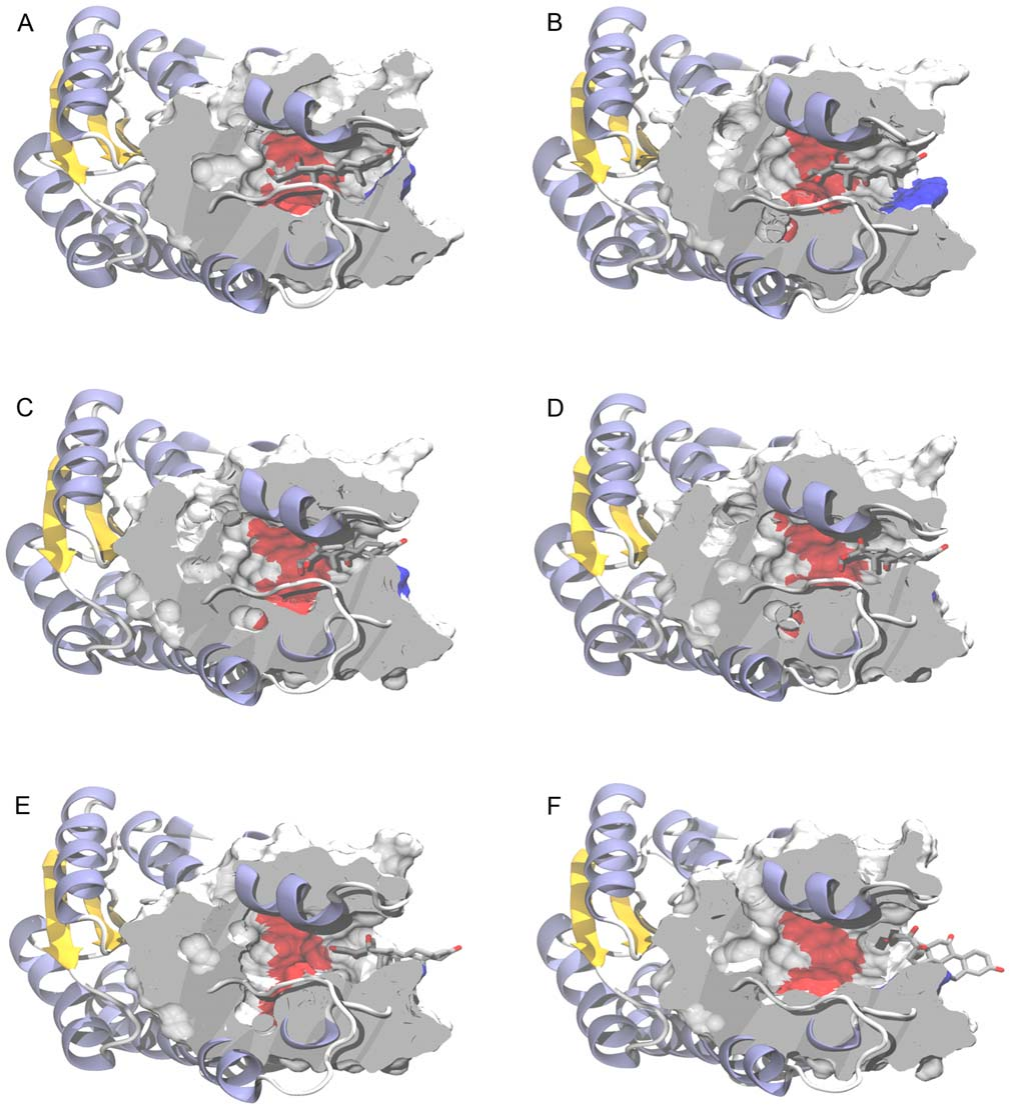
Selected moments of the cortisone path from within the catalytic site to the solvent. Cortisone is depicted as stick model C-coloured white while 11β-HSD-1 is represented as cartoon, coloured according to the secondary structure elements (blue for α helices and yellow for β sheets). Binding site defining residues are highlighted as Connolly surface. To help interpretation the convolution of the Connolly surfaces belonging to S170, Y183 and the NADPH nicotinamide ring are coloured in red, while that of Y177 is coloured in blue. Configurations (A) to (F) are labelled according to the basins **A** to **F** of the FES reported in Figure 8.

The FES, obtained after ∼100 ns of simulation, when the re-crossing event (*i.e.* cortisone docking) occurred [36], is reported in Figure 8E. Several minima could be identified in the free energy landscape, each of which (labels **A** to **F** in Figure 8E) corresponded to a worth-analysing configuration of the system (Figure 9).

The time evolution of the FES at 20, 40, 60, 80 and 98 ns can be followed in Figure 8 (panels A to E). The landscape could be roughly divided into three important parts (dashed lines in Figure 8E). The first one, from 2 to 10 (in the *S* dimension), corresponds to the motion of cortisone into the catalytic site. The second part, from 10 to 16, describes the configurations soon after the substrate detachment when an almost complete solvation of the active site occurred. The remaining part of the plot illustrates the complete ligand detachment which resulted in the total hydration of the binding site. As pre-announced by the SMD simulations, the cortisone exit path seemed to be characterised by a step-wise switching mechanism between progressively less stabilising protein interaction stations, until a completely detached configuration was achieved.

As one might have expected, the configurations found in proximity of the deepest minimum located approximately at *S* = 4 and *Z* = 1 Å^2^ (basin **A** in Figure 8E), corresponded to the catalytically competent pose (Figure 9A). In this region of the collective variables space, the substrate was subjected to continuous rearrangements seeking favourable interactions, resulting in a wide basin spanning 3 units in *S* and 2 units in *Z*. Here, the stabilising interactions of cortisone with S170 and Y183 were repeatedly lost and gained while both the hydrophobic contacts with Y177 and some polar interactions with bridging water molecules at the bottom of the cavity were firmly kept. The relatively large extension of this basing along the *Z* dimension can be mainly explained in terms of rotational entropic states sampled along the major axis of the cortisone, contributing to the thermal stabilisation of the binding configuration. The next basin (basin **B** in Figure 8E) was characterised by a switching of interactions of the molecule with respect to the protein frame. Indeed, whereas the previous basin was primarily stabilised by a set of interactions corresponding to the catalytic configuration adopted by cortisone in the binding pocket, the basin **B** represented a non-catalytically competent, albeit considerably stable state (only 3 kcal mol^-1^ less than basin **A**). The main interactions found were attributed to the flexible tail of cortisone with both the NADPH ribose 2′-OH and Y183-OH, and to the 11-keto function with the backbone nitrogen of residue L171 which forms (along with residues 172 and 173) a 3_10_ helix located in proximity to the entrance of the cavity. During this stage of the undocking pathway, the substrate lost the direct interactions with the highly charged ß-phosphate, and this could explain in retrospect the rupture force experienced at about 1.0 Å of extension observed in most of the SMD simulations. Notably, in spite of a partial opening of Y177 toward the solvent (blue Connolly surface in Figure 9B), this residue seemed not to adopt an effective orientation for the unbinding (average χ1 dihedral angle of Y177 of about 160 degrees compared to the values comprised between 60 and 70 degrees observed in the configurations belonging to basins **C**, **D**, and **E**). Within this minimum, cortisone strongly interacted with Y177 by means of hydrophobic interactions, as it can be clearly inferred by the co-planarity of the steroid A ring and the tyrosine aromatic ring depicted in Figure 9B. The same effect was even more pronounced in the configurations found in basin **C** (Figure 8E), although the effective reorientation of Y177 for the ligand unbinding preluded the first relevant activation barrier for the transition. In basin **C**, a progressive disruption of the interactions with the polar residues at the bottom of the site (red Connolly surface in Figure 9C) took place. At those values of *S* a) the flexible tail of cortisone directly interacted with Y177 and S170, b) the 11-keto moiety preserved the previously established contacts with residue L171, and c) the 3-keto group began to sense the presence of water. The same considerations could be applied to basin **D**, whose distance from the minimum **C** is mostly due to the *Z* component. We hypothesise here that basins **C** and **D**, taken together, may constitute a wide metastable state representing a second interaction station required do drive the cortisone undocking from the catalytic site.

**Figure 10:**
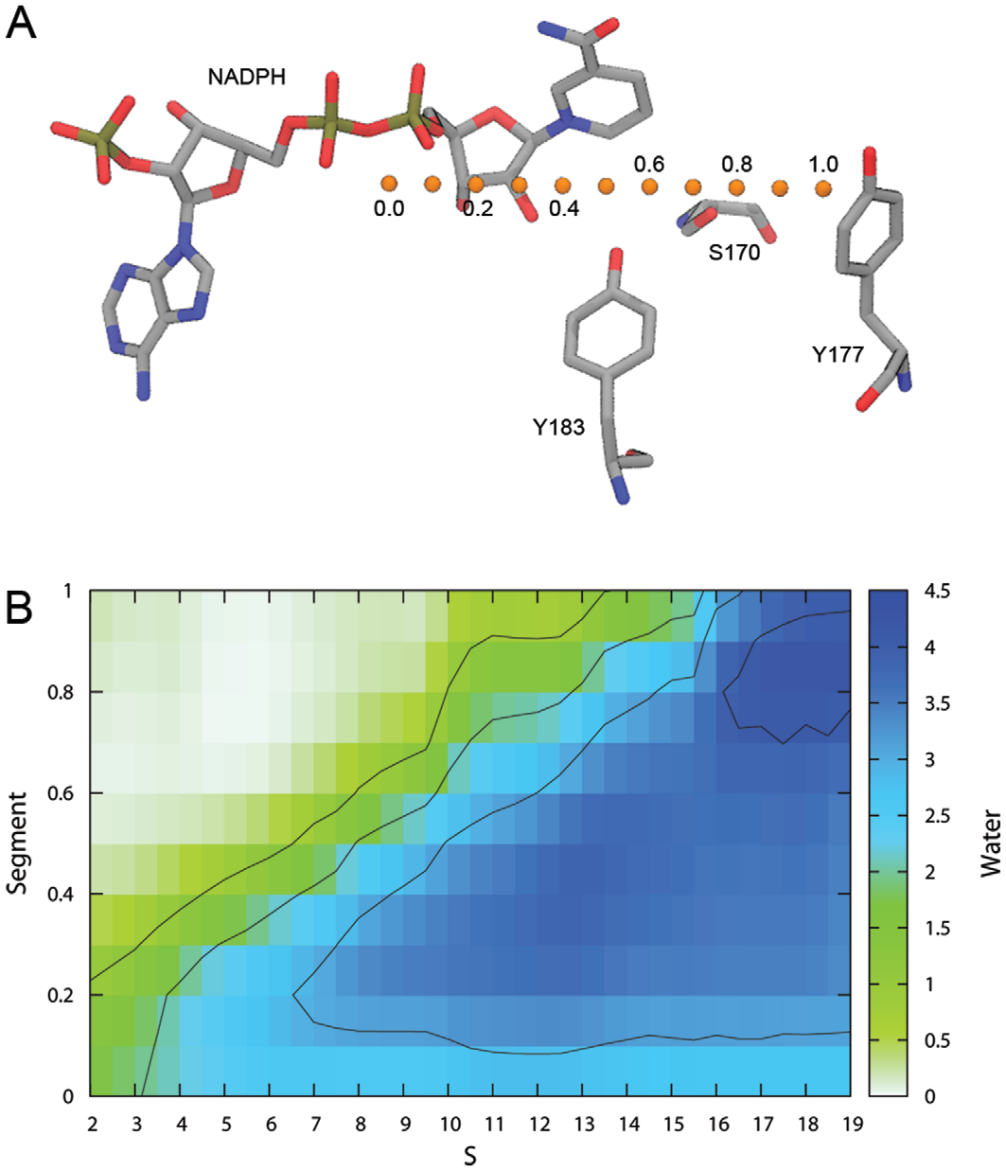
Analysis of the binding site solvation. (A) Graphical representation of the points belonging to the main axis of the binding site, where the average water coordination, during the metadynamics simulation, was calculated (the so-called “Segment” in (B)). (B) Water coordination calculated on the major axis of the catalytic site as function of S. Iso-lines separation is of one water molecule.

Once all the interactions established within the site were lost (Figure 9E) cortisone was free to trespass the Y177 boundary, seeking the next minimum (basin **E** in Figure 8E), found after an energy barrier of approximately 9 kcal mol^−1^ with respect to basin **A**. Most of the ligand-protein stabilisation here was due to the B and C rings of cortisone sliding onto the aromatic part of Y177. At this point, located at *S* values of 13, the binding site, deprived of its substrate, resulted almost completely solvated (see Figure 10 for a complete description of the cavity solvation process).

The relative stability of basin **E** (about 7 or 8 kcal mol^−1^ from basin **A**), and its location in proximity of the main activation barrier may support the picture of a metastable state required for the proper substrate recognition by the enzyme. The last minimum could be identified at *S* = 18 and *Z* = 6. As it can be gathered from Figure 9F, approximately 90% of the ligand was outside the protein cavity, completely exposed to the solvent. At this stage no specific intermolecular interactions with the protein could be detected. It took as much as 60 ns for the undocking event to take place. Then the system kept evolving onto the bias imposed by the undocking-generated FES. Under this circumstance the re-docking event occurred after approximately 40 ns, for a grand total of 98 ns of metadynamics simulation. The ΔG of binding calculated as the free energy difference between basins A and F was 6 kcal mol^−1^. This value is compatible with the formation of the Michaelis complex in light of both the experimentally found *K_m_* value of 9.6 µM and the limited catalytic efficiency (*k_cat_*/*K_m_* = 2.6×10^5^ M^−1^ min^−1^) of the cortisone-to-cortisol conversion [7]. As it can be seen in Figure 8F, where the progression along the reference path is plotted as function of the simulation time, after the undocking event, the substrate tried to re-enter the active site almost immediately. However the relevant barriers could be overcome only after several attempts and approximately 30 ns of simulation. At this stage, after 90 ns of metadynamics, the added bias allowed a smooth entrance of cortisone into the catalytic site which occurred in about 10 ns.

As reported by some of us, the S228-P237 loop is supposed to be important to tune the substrate specificity at superfamily level [32], [33]. Indeed, during the simulations, that loop resulted the most movable part of the system. Although this behaviour was already evident during the MD and SMD runs (see Figure 5), it should be remarked that it was not explicitly included as a separate collective variable. The cortisone exit was accompanied by a concerted and unbiased motion of that loop with a consequent subtle rearrangement of the 2 α helices connected to it (T220-V227 and K238-L251).

One of the most problematic aspects of simulations that deal with protein-ligand binding events is related to the presence of solvent molecules in the active site, which must be displaced upon binding. In our study, the gradual solvation and desolvation of the cavity was possible thanks to the presence of a small entrance, located in proximity of the NADPH cofactor, defined by K44, N123, and T222 (see Figure 2). In Figure 10B, the water coordination to the enzyme during the metadynamics simulation is plotted as function of *S* and a segment of unitary length describing the major axis of the binding site (see Methods for details). The origin of the segment (value of 0.0, see Figure 10A) is approximately located at the bottom of the gorge, that is in the direction of NADPH, whereas the end (value of 1.0) is found in correspondence of Y177 at the top of the cavity, The image shows that, at low values of *S*, the pocket was almost completely desolvated, while, concomitant to the substrate move towards Y177 (*i.e.* for higher values of *S*), a steady hydration was observed. Notably, at *S* = 7, where the minimum **B** is located, an average number of more than two water molecules was found in correspondence of a segment value of about 0.4, indicating a full detachment of the flexible polar tail of the cortisone from the β-phosphate of NADPH (see Figure 10B). Conversely, the residues directly involved in catalysis (S170 and Y183) achieved a substantial degree of solvation only after reaching the basin **E** which is located approximately at *S* = 13 (see Figure 10A and Figure 10B, segment values of 0.8).

When the molecule was completely disengaged from the enzyme, solvent molecules readily took its place by occupying entirely the catalytic site (*i.e. S* = 18). This feature, already highlighted during the SMD trajectories, is supposedly an essential structural aspect for the enzyme functioning that allows a correct turnover of water molecules upon cortisone binding/unbinding. It is worth remarking here that, in our simulation, this functional characteristic of the enzyme was observed both during the undocking/solvation and the docking/desolvation events. This can be taken as a direct evidence of the good *health* of the simulated system, which could adequately sample all the degrees of freedom relevant to the phenomena.

### Y177A mutant

**Figure 11:**
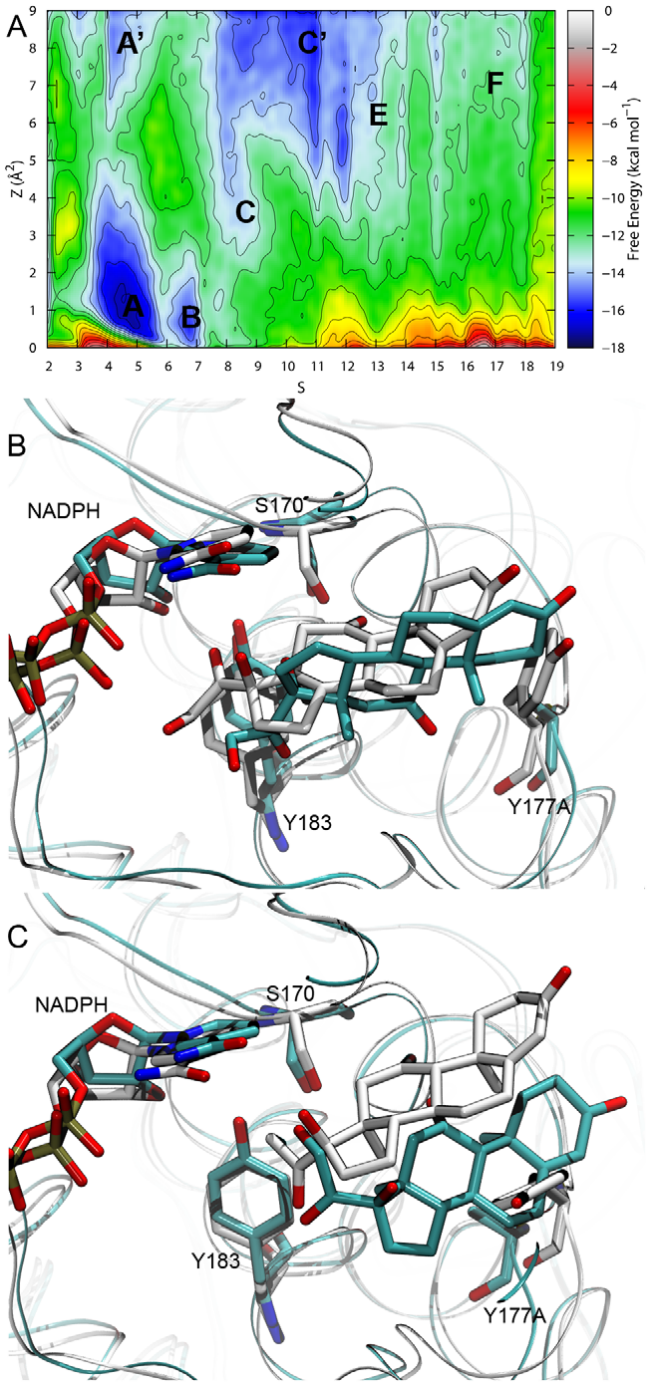
Cortisone binding process for the Y177A mutant. (A) Free energy surface as reconstructed after 108 ns of simulation. Iso-lines separation is 1 kcal mol^−1^. Worth-analysing minima are highlighted (labels **A, A', B, C, C', E** and **F**). (B) Comparison between the configurations found in basins A and A' (WT and Y177A mutant, respectively). (C) Comparison between the configurations found in basins C and C' (WT and Y177A mutant, respectively). The system involving the WT enzyme is highlighted C-coloured white while the one involving the Y177A mutant is depicted C-coloured cyan. The protein is represented as ribbons.

To achieve a superior understanding of the cortisone unbinding mechanism and energetic, additional metadynamics simulations involving the Y177A mutant were carried out. In Figure 11A, the FES obtained after about 110 ns of simulation is shown. Several correspondences between the FES profiles reconstructed for the wild type (WT) and Y177A mutant could be observed, especially at *S* values up to 8 units, where many of the occurring binding interactions were retained by the two systems. However, even in this portion of the PCVs space, the distinctive effect of the mutation is appreciable when the shapes of the corresponding basins **A** (*i.e.* where the catalytic competent configurations took place) are compared. While in the WT enzyme, basin **A** was localised at *Z* values close to zero (see Figure 8), in the Y177A mutant the same basin (*S* ranging approximately from 3.5 to 6) was significantly extended along the *Z* coordinate. Most importantly, at high *Z* values (*Z*>5.5) a secondary basin was found (labelled as **A'** in Figure 11A). A careful analysis of the trajectory revealed that this ancillary free energy well was characterised by a catalytically non-competent configuration of the substrate. As it can be seen in Figure 11B, the substrate was considerably rotated along its principal inertia axis, preventing a productive arrangement of the groups involved in the catalysis. While basins **B** and **C** were found similar in the two systems, distinctive features of the FES reconstructed for the Y177A mutant are the absence of basin **D**, and the presence of a deep and large basin at *S* values spanning approximately from 8 to 11, and *Z* greater than 5 units (labelled as **C'** in Figure 11A). Such basin was less stable than basin **A** only of about 1 kcal mol^−1^. In representative configurations belonging to this basin, the cortisone was found detached from the polar area of the binding site (NADPH; Y183 and S170, see Figure 11C) with a varying number of interposed water molecules. Beyond that basin, a comparison between the FES of WT and that of the mutant becomes not viable due to the expected significantly different exit paths followed by the substrate. Even though a free energy well analogous to basin **E** might be found at *S* values ranging from 13 to 14 (see Figure 11A), basin **F** is more difficult to localise, being the free energy landscape almost flat in that region. Notably, the energy barrier for the unbinding process in the mutant was lowered of about 4.5 kcal mol^−1^ with respect to the WT, and the free energy of binding could be estimated to be approximately 4 kcal mol^−1^.

In summary, the increase in *K_m_* value experimentally obtained for the Y177A mutant can be imputed to two concomitant destabilising mechanisms. On the one hand, the mutation lowers the free energy difference of binding for the substrate. On the other hand, once a bound configuration of the cortisone is reached, the probability to retain a catalytically competent configuration is greatly diminished in comparison to the WT enzyme. Such an increase in configurational space, found in the Y177A mutant, reflects the possibility to visit a larger number of configurations. Conversely, this behaviour is suppressed in the case of the WT as a net result of the hydrophobic interactions established between Y177 and the ring A of the substrate.

## Discussion

The process of molecular recognition, at the basis of life itself, is multifaceted and extremely hard to understand. What are the driving forces that come into play? How can enzymes pinpoint partner ligands, either being substrates or inhibitors, within a crowd of possible candidates? What are the thermodynamics consequences of an interaction between a pair of molecules? Even though possible answers can be given based on experimental evidences, the need to have a better understanding on what goes on at a molecular level has prompted researchers, on the one hand, to develop improved force field descriptions [37], [38], [39], [40] to better model molecular interactions, and on the other hand, to deal with physics based computational methods which allow for the accurate mapping of the underpinning energy landscape. Without disregarding the importance of using cutting edge potential energy functions, whenever the aim of a work is mostly focussed on a semi-quantitative description of a biological event, a satisfactory picture can be generally achieved also with the use of simpler and widespread, additive force fields with the concurrent benefit of saving significant computational time. Concerning the methodologies employed to enhance the sampling, among other methods, metadynamics has recently shown promising results when challenged with complex biological problems, typically characterised by several degrees of freedom [27], [28], [41], [42]. In this context, metadynamics was applied for the first time to study how an enzyme interrelates with its natural substrate.

During the simulations, several mechanistic details of 11β-HSD-1 became evident. In particular, a possible reason why the enzyme needs to be at least dimeric to be functional could be found in the stabilisation of the catalytic site, which would be greatly affected in the absence of one of the chain. Another peculiar feature of 11β-HSD-1 was given by the presence of K44, N123, and T222 at the bottom of the cavity. Those 3 residues defined a small functional back-door that allowed water to enter or exit, making it easier for the substrate to leave or occupy the site. If the solvent could not at ease leave the cleft upon substrate docking, the binding event would be difficult to achieve. *Vice versa*, during the cortisone unbinding, water molecules should promptly replace the ligand to prevent vacuum formation that would hamper the undocking process. Such hypothesis, although lacking a direct experimental validation, offers an attractive way to account for the solvent displacement upon substrate binding. Also the catalytic hypothesis was fully confirmed by the computational simulations since all the functional groups supposedly involved in the reaction were stably within reasonable cutoffs of distances for the reaction to occur. We note that the potential interactions at the catalytic site have been already modelled by Hosfield and colleagues [7]. However, the authors studied the complex in a static way, placing the substrate in the active site and energy minimising the resulting structure. In this sense, our work could be seen as an evolution of the static model there proposed. The last major finding in our study was about the role played by Y177 in substrate recognition and binding. The theory proposed by Wang and collaborators [14] that addressed Y177 as an unlikely H-bond donor for cortisone found full support in our results. The retention of activity by the Y177F mutant and the loss of activity by Y177A and Y177Q can be more easily explained considering the fundamental job of this residue during the simulated binding events. The comparative simulation of the Y177A mutant clearly highlighted the fundamental role of the tyrosine by means of stabilising interactions with the A ring of the substrate. In fact, as a consequence of the mutation, a lower difference in free energy was recorded and the presence of alternative basins in the FES of the mutant was observed.

In conclusion, the cortisone unbinding and binding to 11β-HSD-1 were re-enacted by means of a PCVs metadynamics simulation. The method allowed us to obtain an trustworthy mapping of the FES of binding which helped to put experimental data into context and to gain unprecedented insights on the enzyme functioning. The work presented here has remarked the potential of computational techniques to capture the anharmonic and multiwell interplay between molecules. Developments in the field are expected to bring us closer to a full understanding of the protein-ligand recognition and binding processes.

## Methods

### Selection of structural data

Among the several X-ray structures of 11β-HSD-1 available at the wwPDB [12] the one in complex with NADPH and carbenoxolone, a tight binder of the enzyme, solved at 2.11 Å, was selected for our investigations (PDB ID: 2BEL). Such a choice was, among other aspects (*e.g.* overall quality of the deposited data), driven by the fact that the structural resemblance between carbenoxolone, a synthetic derivative of glycyrrhizinic acid, and cortisone was likely to grant a reliable starting guess of the protein-substrate arrangement upon docking [43].

### Docking simulations

The model structure 2BEL was imported in Maestro [44] and treated with the protein preparation wizard. Chain A and B were retained. All water molecules were removed as well as ions. Missing side-chains and hydrogen atoms were added to the model which was energy minimised using the default settings. Contextually, a 3D model of cortisone was manually prepared in Maestro. The docking simulation was performed using Glide [45] in Standard Precision (SP) mode. In order to soften the potential of non-polar parts of the substrate, the vdW radii of atoms with partial atomic charge lower than 0.15 were scaled by a factor of 0.8. The docking volumes were two boxes centred at the geometric centres of each carbenoxolone molecules, spanning 35 Å in each direction. At the end of the run, 10 poses per monomer were retained and energy-ranked. For the Y177A mutant, before the protein preparation wizard step the residue Y177 was mutated into alanine.

### Setup of the system and Molecular Dynamics

MD simulations were performed with the NAMD-2.6 [46] program, and the Amber force field parm99SB [47] was employed throughout. The Ryde parameters for the NADPH cofactor were used [48], whereas the cortisone was treated employing the General Amber Force Field [49] for organic molecules along with partial charges derived through the RESP procedure [50], [51] from the electrostatic potential calculated with the Gaussian03 package [52] at the HF/6-31G(d)//HF/6-31G(d) level of theory. Two sets of simulations were carried out: one involving the monomer A only, and the other considering the dimer. In both cases, the protein was introduced in a orthorhombic box of TIP3P water molecules [53] with the cell margin separated of 8 Å from the solute in each dimension. The electroneutrality of the system was achieved by uniformly rescaling the charge excess to zero onto the atoms of the protein [54]. Preliminary simulations pointed out a remarkable protein instability occurring at the residual C-terminal helix (residues 260 to 276, data not shown), that is the putative monotopic domain interacting with the membrane [7]. Not surprisingly, this region is associated to a high Beta factor in most of the 11-βHSD crystallographic structures and, due to its proximity to the entrance of the catalytic site. In order to avoid artefacts during the substrate exit, the C-terminal helix was deleted from the structures employed in the current study.

All the simulations were performed by means of Langevin dynamics using a damping coefficient of 5 ps^−1^ and a uniform integration time step of 2 fs. Bonds involving hydrogen atoms were restrained to their equilibrium geometry with the SHAKE algorithm [55]. Short range non-bonded interactions were treated using a cutoff radius of 12 Å as well as a zero switching function active at distances larger than 10 Å. A neighbour list having a radius of 13.5 Å was used and updated every 10 integration time steps. Periodic boundary conditions were employed, and long range electrostatics was estimated by means of the Particle-Mesh Ewald method [56] using a grid spacing of less than 1 Å in each dimension and a fourth order interpolation scheme. The two sets of simulations followed the same equilibration protocol. First the systems were gradually heated to the target temperature of 300 K during 150 ps of MD performed in the canonical ensemble (*i. e.* constant number of particles, volume, and temperature, NVT). During this stage the restraints initially applied on the backbone atoms of the protein were smoothly released. Then, the sampling was switched to the isothermal-isobaric ensemble (*i. e.* constant number of particles, pressure, and temperature, NPT) and the dynamics was continued for further 200 ps using the Langevin piston method [57] as implemented in the NAMD code [46] at the target pressure of 1 bar. At this stage, residual restraints acting on the Cα atoms belonging to the conserved secondary structure of the protein were retained. Finally, 5 ns of production were performed in the NPT ensemble on the fully unrestrained system.

### Steered Molecular Dynamics, path parameterization and metadynamics

SMD and metadynamics simulations were performed using NAMD-2.6 [46] patched with PLUMED-1.0 [58] by explicitly applying forces onto the A monomer only, while the whole dimeric protein was considered in the system definition. The monomer B was treated in complex with a molecule of cortisone, as obtained through the docking runs. The starting configuration for these simulations was prepared extending by 1 ns the equilibrium production run in the canonical ensemble by using the previously acquired averaged cell dimensions.

Constant velocity SMD simulations were performed in the NVT ensemble by using a radial reaction coordinate defined as the distance between the centre of mass (COM) of the ring A of the cortisone and the COM of the Cα atoms of a set of residues located at the bottom of the catalytic pocket (amino acids: 45 to 47, 120 to 122 and 220 to 223). Trial simulations were carried out in order to optimise both the selection of the atoms involved in the reaction coordinate and the pulling regime. A force constant of 20 kcal mol^−2^ and a speed of 0.005 Å ps^−1^ turned out to be a reasonable compromise between accuracy of the detected events and the computational load needed to ensure the loss of the leading interactions of the cortisone with the protein (extension of 7.5 Å). Five realisations of the unbinding process (1.5 ns each) were collected starting by the same configuration and by re-initializing the starting velocities. The trajectory with the smoothest force profile (run 5, Figure 7) extended up to 3 ns was then used as input for the definition of the unbinding path.

As in any method that attempts to estimate the free energy profile of a rare event based on a dimensionality reduction, the reliability of the results achieved by means of metadynamics simulations are strongly dependent upon the choice of the collective variables. In this study, to treat at best the unbinding of the cortisone from the catalytic cleft, a path collective variables (PCVs) [59] approach was employed. According to the PCVs framework, a pair of orthogonal variables designed to map the configurational space (**R**) of the atoms involved in their definition along the investigated transition were used. In particular, the *S* variable describes the *progression* along a putative path, whereas *Z* is a measure of the *distance* from it [59]. They are defined as:
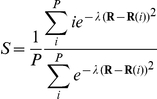


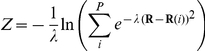



where *P* is the total number of configurations employed in the definition of the guess path (hereafter referred to as frameset), and *λ* is a tuneable parameter controlling the smoothness of the *S* function. The distances between the configurations explored in time and those constituting the frameset were calculated using the MSD metric [60]. In particular, the Cα atoms belonging to the β-barrel core of the protein were used as reference frame for the least square fit superposition due to the stability shown by these residues during the 5 ns of plain MD (see the RMSF of the β-barrel regions in Figure 5). Conversely, the distance after superposition was measured using all the heavy atoms of the cortisone. Since the entrance of the catalytic site is mainly constituted by flexible and easily adapting loop regions, no atoms of the protein were explicitly included for the measurement of the PCVs distances. The frameset was then parameterized by extracting 18 configurations from the run 5 of SMD. This procedure was performed in an automate way by satisfying the requirement to provide a set of neighbour configurations as evenly distributed as possible so to ensure an optimal mapping of the transition along the *S* variable. The resulting frameset was eventually characterised by an average spacing of 1.1 Å, and by a *λ* value of 1.94.

The continuous [61] and direct [25] formulation of metadynamics [24] as implemented in the PLUMED-1.0 plugin [58] was used. In particular, a Gaussian deposition rate of 0.1 kcal mol^−1^ ps^−1^ was used along with a Gaussian width of 0.15 units in the *S* dimension and 0.20 Å^2^ in *Z*. The metadynamics simulation was conducted in the canonical ensemble until a fully converged free energy surface was obtained, that is until the re-crossing event, corresponding to the return of the cortisone to the catalytic site (completed after about 100 ns of simulation) was achieved.

The same PCVs' frameset optimised for the WT enzyme along with the same simulation parameters were employed to perform the metadynamics on the Y177A mutant. In this case, the simulation was interrupted after about 110 ns, when the re-crossing event occurred.

Most of the data analysis was performed either by means of the utility drivers distributed with PLUMED-1.0 [58] or by using the Tcl scripting interpreter embedded in VMD [62]. Cluster analysis on the configurations belonging to the different basins identified on the free energy landscape was performed with Gromacs-4.0.5 [63] using the single linkage algorithm over the Cα carbons of the protein and the cortisone heavy atoms with a distance cutoff threshold of 0.7 Å. The water coordination along the major axis of the catalytic pocket was calculated by properly modifying the PLUMED collective variable deputed to estimate the coordination number around an atom. The major axis of the catalytic pocket was defined by a segment constructed by selecting a convenient subset of protein atoms located in proximity of the bottom and the top of the binding site whose relative displacement experienced during the metadynamics simulation was negligible. The segment mimicking the catalytic pocket major axis was then arbitrarily divided in 10 slices to achieve a total of 11 points (values of 0.0, 0.1, 0.2, 0.3, 0.4, 0.5, 0.6, 0.7, 0.8, 0.9, 1.0 of the segment axis in Figure 10B).

The number of water molecules located in proximity to each of the 11 points of the segment was estimated as [58]:
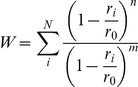



where *r_i_* is the distance between the oxygen atom belonging to the *i^th^* water molecule and the considered point, *r_0_* was set to 3.5 Å, *n* = 6 and *m* = 16. The summation runs over all the water molecules of the system. The number of water molecules calculated in correspondence of each point of the segment was averaged along the whole *Z* dimension and over slices of 0.5 units of *S*.
